# Identification of a novel deletion in the MMAA gene in two Iranian siblings with vitamin B12-responsive methylmalonic acidemia

**DOI:** 10.1186/s11658-016-0005-1

**Published:** 2016-07-28

**Authors:** Fatemeh Keyfi, Mohammad Reza Abbaszadegan, Arndt Rolfs, Slobodanka Orolicki, Morteza Moghaddassian, Abdolreza Varasteh

**Affiliations:** 1 Allergy Research Center, Mashhad University of Medical Sciences, Mashhad, Iran; 2 Immunology Research Center, School of Medicine, Mashhad University of Medical Sciences, Mashhad, Iran; 3 Division of Human Genetics, Immunology Research Center, Avicenna Research Institute, Mashhad University of Medical Sciences, Mashhad, Iran; 4 Pardis Clinical and Genetic Laboratory, Mashhad, Iran; 5 Director of the Albrecht Kossel Institute for Neuroregeneration, University of Rostock, Rostock, Germany; 6 Chief Medical Director, Centogene AG, Rostock, Germany; 7 Clinical and medical scientist, Centogene AG, Rostock, Germany

**Keywords:** Mutation analysis, MMAA gene, Biochemical analysis, Methylmalonic acidemia, Vitamin B12, Novel deletion, Cobalamin, Structural analysis

## Abstract

**Background:**

Adenosylcobalamin (vitamin B12) is a coenzyme required for the activity of methylmalonyl-CoA mutase. Defects in this enzyme are a cause of methylmalonic acidemia (MMA). Methylmalonic acidemia, cblA type, is an inborn error of vitamin B12 metabolism that occurs due to mutations in the MMAA gene. MMAA encodes the enzyme which is involved in translocation of cobalamin into the mitochondria.

**Methods:**

One family with two MMA-affected children, one unaffected child, and their parents were studied. The two affected children were diagnosed by urine organic acid analysis using gas chromatography-mass spectrometry. MMAA was analyzed by PCR and sequencing of its coding region.

**Results:**

A homozygous deletion in exon 4 of MMAA, c.674delA, was found in both affected children. This deletion causes a nucleotide frame shift resulting in a change from asparagine to methionine at amino acid 225 (p.N225M) and a truncated protein which loses the ArgK conserved domain site. mRNA expression analysis of MMAA confirmed these results.

**Conclusion:**

We demonstrate that the deletion in exon 4 of the MMAA gene (c.674 delA) is a pathogenic allele via a nucleotide frame shift resulting in a stop codon and termination of protein synthesis 38 nucleotides (12 amino acids) downstream of the deletion.

## Background

Vitamin B12, also called cobalamin (Cbl), is a water-soluble vitamin with an important role in brain and nervous system function. It is involved in the metabolism of fatty and amino acids and is an essential vitamin that must be provided in the diet [1]. Inborn errors of cobalamin metabolism cause dysfunction in two essential coenzymes, adenosylcobalamin (AdoCbl) and methylcobalamin (MeCbl). AdoCbl is required for activity of the mitochondrial enzyme methylmalonyl-CoA mutase (MCM, EC 5.3.99.2), which catalyzes the conversion of methylmalonyl-CoA to succinyl-CoA during catabolism of branched-chain amino acids and odd-chain fatty acids. Disorders that impair synthesis of AdoCbl cause accumulation of methylmalonic acid in urine and blood due to decreased activity of methylmalonyl-CoA mutase, which leads to methylmalonic acidemia (MMA) [2].

Individuals affected with this disorder present with lethargy, failure to thrive, recurrent vomiting, dehydration, respiratory distress, and hypotonia in the first year of life [2].

The gene responsible for cblA has been identified as MMAA [3]. MMAA is located on chromosome 4q31.1–2, and consists of seven exons that encode a protein containing 418 amino acids. Two different enzymatic functions have been identified for the MMAA gene product: a role in vitamin B12 transport into the mitochondria, and the conservation or re-activation of MCM [4]. In this study, we report the clinical, biochemical, and genetic defects and structural analysis of MMAA in one Iranian family with isolated MMA.We demonstrate that this novel deletion in exon 4 of MMAA is a pathogenic allele that results in a premature stop codon and subsequent truncated protein and the loss of the important binding sites of the protein due to loss of the ArgK (PF03308) conserved domain.

## Materials and methods

### Patients and MMA diagnosis

One family with two MMA-affected children, one unaffected child, and their parents were studied. A daughter who died five days after birth was not assessed. The two affected children were diagnosed after presenting with clinical symptoms and high levels of methylmalonic acid by urine organic acid analysis. All procedures followed were in accordance with the ethical standards of the committee on human experimentation of MUMS (Mashhad University of Medical Science). Informed consent was obtained from all subjects for being included in the study.

### Mutation and mRNA expression analysis

DNA was extracted from the family members’ blood samples using the salting-out method [5]. MMAA exon 4 and flanking sequences were amplified by PCR using primers designed with Beacon Designer (forward: 5′-AGGAACTGGCTGATAATTGA-3′, reverse: 5′-AGGGAGTATGGGATATCTTG-3′). For expression analysis, RNA was isolated using Trizol reagent (Invitrogen). RT-PCR was performed and the coding sequence was amplified using primers designed with Beacon Designer (forward: 5′-TACCACAGAGAACAAGAACAATCA-3′, reverse: 5′-GCACAATCAAGTCTCCATCAG-3′). At least two PCR products were directly sequenced and products of the sequencing reaction were analyzed using DNA Sequencher 4.7.

### Structural and functional analysis

The novel mutation (p.N225M) was considered for the purpose of structural modeling in order to completely understand the vast structural damages caused by truncation of the protein after the occurrence of the deduced novel mutation in comparison with the normal structure. Although the protein loses a large part of its structure, this structural modeling would help to better understand the vast dysfunctioning of the mutated protein by visually analyzing it. To accomplish it, the template most similar to the deduced normal protein with 95.7 % sequence similarity (Swiss Model Template ID (SMTL ID): 2www.1) was chosen and used to build the tertiary structure of the normal and mutated proteins by using the online Swiss-Prot server for automated modeling [6]. The 2www.1 structure has been solved to 2.64 Å using the X-ray diffraction method [7]. The structures were then considered for the purpose of the energy minimization task using ZMM software. ZMM software uses amber-all-atom force field with the cut-off distance of 10 Å to minimize the conformational energy in the space of generalized coordinates [8]. Low energy conformation was reached after 100 sequential tasks of energy minimization failed to actually enhance the quality of the assembled structures [9].

The resulting models were finally analyzed in terms of essential accuracy aspects such as G-Factor, bond length and bond angles by the help of PROCHECK [10] and WHAT-IF [11] online programs for of which all the results satisfied the important criteria. Moreover, most of the residues were inside of the favorable regions of the Ramachandran map.

In addition to revealing the structural defects, the effects of this novel mutation (p.N225M) on the function of the deduced protein was further investigated by considering its mutational role on the function of the conserved ArgK (PF03308) domain which starts at LUE p.101 and ends at THR p.384.

## Results and discussion

### Clinical symptoms and biochemical findings

**Fig. 1:**
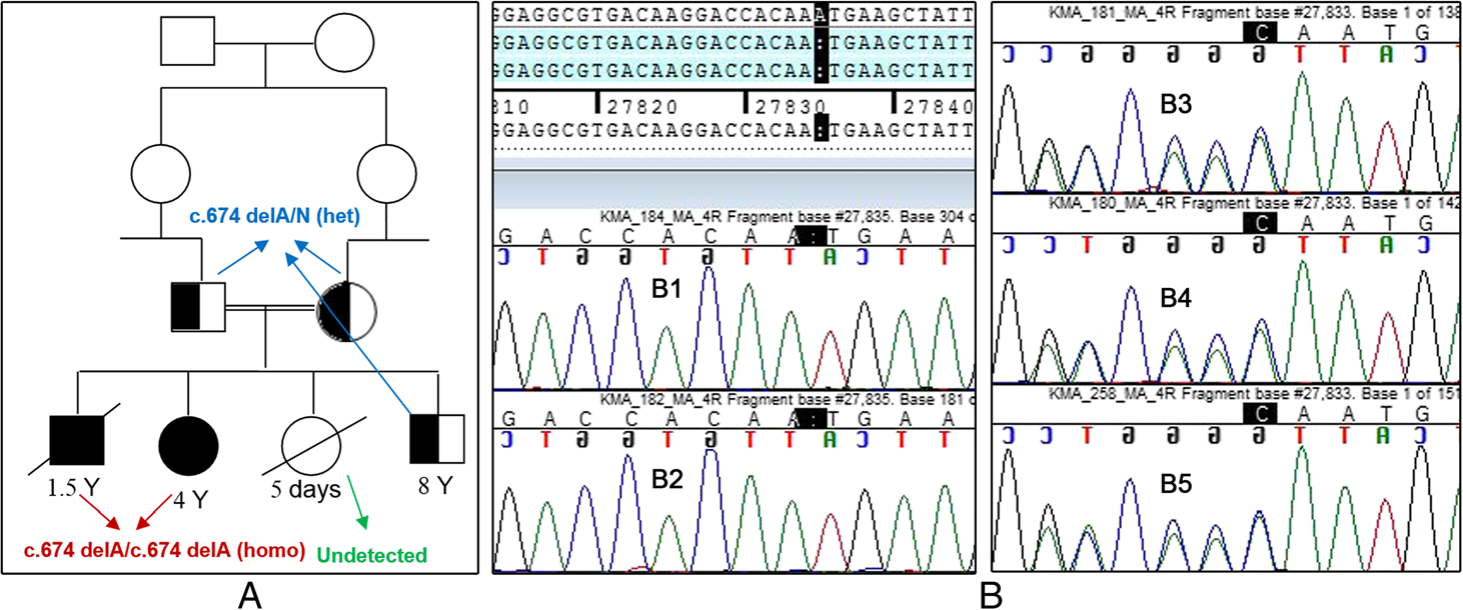
Family pedigree and DNA sequence chromatograms of family members (**a**) Family pedigree and mutation analysis for exon 4 of MMAA (**b**) A DNA sequence chromatogram of the family members (*B1*) DNA sequence chromatogram of the affected daughter (4 years old) with the homozygous c.674delA mutation (*B2*) DNA sequence chromatogram of the affected son (1.5 years old) with the homozygous c.674delA mutation (*B3*) DNA sequence chromatogram of the father with the heterozygous c.674delA mutation (*B4*) DNA sequence chromatogram of the mother with the heterozygous c.674delA mutation (*B5*) DNA sequence chromatogram of the unaffected son with the heterozygous c.674delA mutation

This novel deletion was identified in two children: a 4-year-old girl with metabolic problems, and her 1.5-year-old brother. Both children had signs and symptoms of MMA. The family pedigree is shown in Fig. 1a. The clinical symptoms and biochemical findings of the two affected children are presented below:

The girl, who was a product of a consanguineous marriage, was born at term following a normal pregnancy. She was admitted to the hospital at 2 months of age with a history of persistent vomiting and progressive lethargy. Pyruvate was 1.49 mg/dL (normal, 0.3–0.7), ammonia was 197.60 mmol/L (normal, 64–107), lactate was 42.1 mg/dL (normal, 0 to 20), venous pH was 7.48 (normal, 7.35–7.45), and bicarbonate was 21.9 mmol/L (normal, 22–28). Tandem mass spectrometry analysis showed significant elevation of propionylcarnitine (13 μmol/L), which may be indicative of MMA or propionic acidemia (PA). Urine organic acid analysis indicated abnormally high excretion of methylmalonic acids (1250 mmol/mol creatinine). She was diagnosed with MMA and treated with protein restriction, daily intramuscular hydroxycobalamin injections (1.0 mg), Shohl’s solution (4.5 ml/day), benzoate tablets (250 mg/day), L-carnitine (5 ml/day), and metronidazole syrup (4.5 ml/day). At 4 months she presented with lethargy, vomiting, hepatomegaly, hypotonia, anorexia, developmental delay, and failure to thrive. At 10 months she had begun to achieve sufficient weight and showed developmental and hypotonia improvement with protein restriction and L-carnitine and hydroxycobalamin injections. In the third year of treatment, her urinary methylmalonic acid excretion was 12 mmol/mol creatinine, but development and movement were delayed compared with normal individuals.

The boy’s birth history and development in the first year of life were normal and no symptoms of MMA were observed. He was hospitalized at 1.5 years of age for persistent vomiting and infection. Glucose was 250 mg/dL (normal, 70–105), venous pH was 7.07 (normal, 7.35–7.45), bicarbonate was 6.5 mmol/L (normal, 22–28), blood urea was 55 mg/dL, and creatinine was 0.5 mg/dL. He was treated for MMA with L-carnitine, sodium benzoate, and vitamin B12. Dialysis was initiated, but he died on the second day of hospitalization.

### Novel MMAA deletion and mRNA expression analysis

**Fig. 2:**
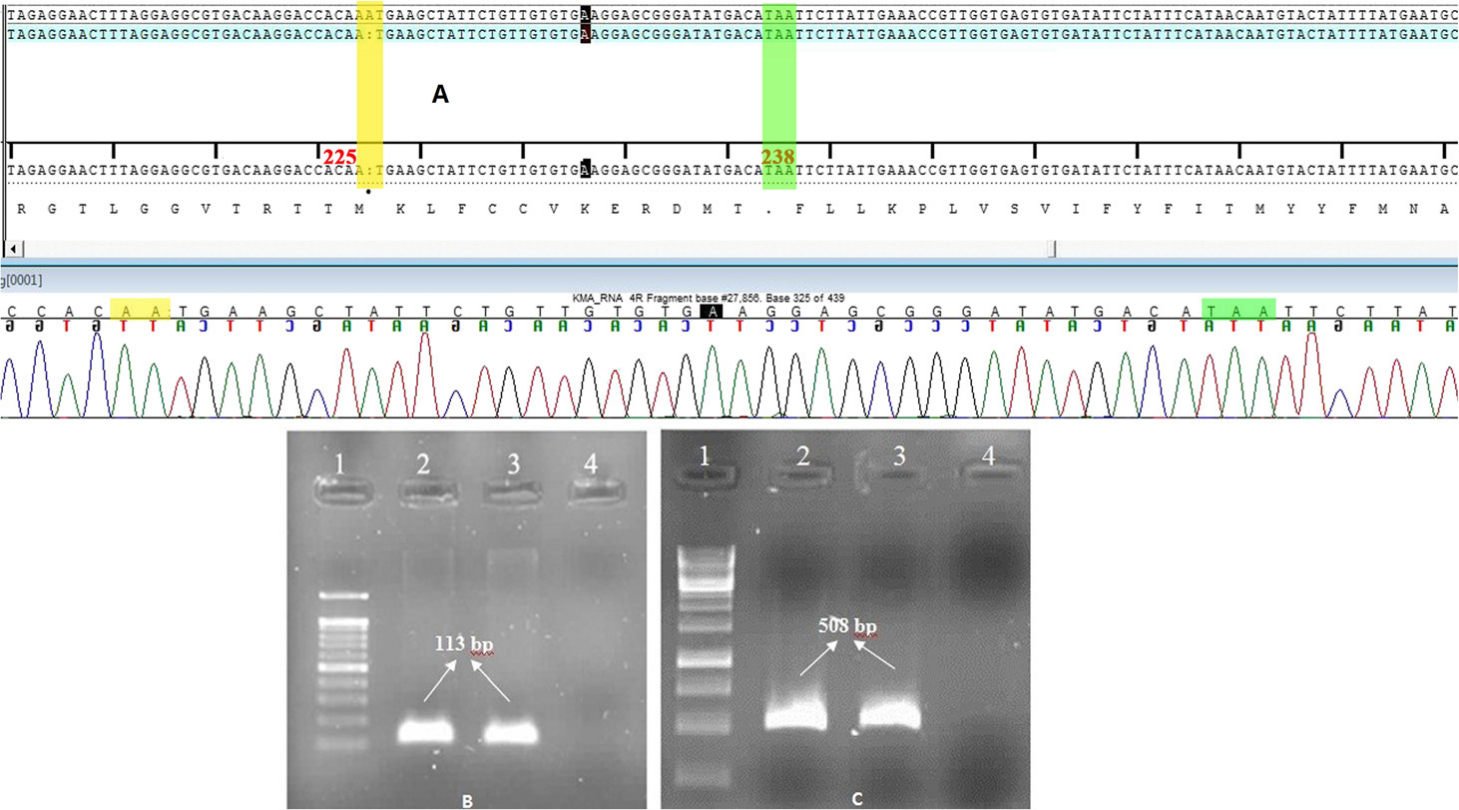
cDNA sequence chromatogram and expression analysis of β-actin and MMAA gene. **a** cDNA sequence chromatogram of MMAA from the affected daughter and the corresponding amino acid sequence, premature TAA stop codon 12 amino acids downstream of the methionine produced by the frame shift (*green*) (**b**) β-actin (size: 113 bp): Lanes 1: 100 bp marker, 2: cDNA from the affected daughter with the c.674delA mutation, 3: cDNA from a normal control, 4: negative control. **c** MMAA, exon 4, size: 508 bp):Lanes 1: 1 kbp marker, 2: cDNA from the affected daughter with the c.674delA mutation, 3: cDNA from a normal control, 4: negative control

The family pedigree and sequence chromatograms for exon 4 of MMAA from the five family members are shown in Fig. 1. A homozygous deletion in exon 4 of MMAA, c.674delA, was found in both the affected son and daughter. An adenine deletion was detected at position c.674 in exon 4. This deletion predicted a nucleotide frame shift and amino acid change from asparagine to methionine at position 225 (p.N225M), resulting in a premature TAA stop codon at position 238 and truncation of the protein 12 amino acids downstream of the methionine produced by the frame shift. We confirmed the mutation’s homozygosity through analyses of the parents’ and unaffected son’s samples. We found one heterozygous variant in exon 4 of MMAA, c.674delA. This mutation has been recorded in the GenBank database with accession number **KR026958**. These results, from two affected and one unaffected siblings and their parents, confirmed the diagnosis of cblA-deficient MMA. MMAA mRNA expression analysis identified the expected truncated message. The mRNA expression analysis and sequencing chromatogram of expression analysis are shown in Fig. 2. The premature TAA stop codon 12 amino acids downstream of the methionine produced by the frame shift are shown in this figure.

### Mutational effects on the structural defects and dysfunctioning of the protein

**Fig. 3:**
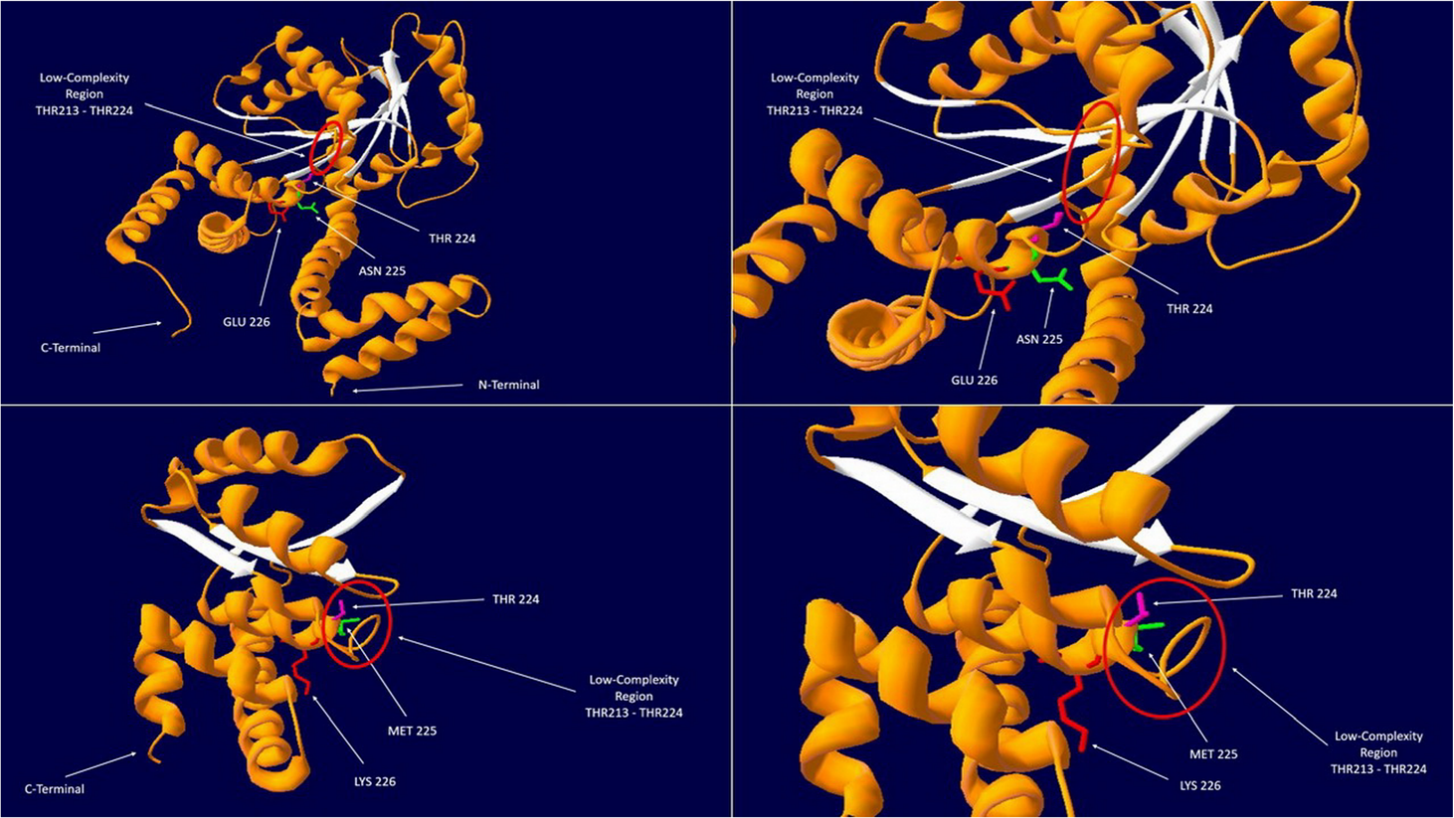
Whole view of the normal protein MMAA by the use of 2www structure (*above left*); close view of the normal protein MMAA (*above right*); whole view of the novel mutated MMAA protein (p.N225M) by the use of 2www structure (*below left*); close view of the novel mutated protein MMAA (*below right*). *Red circles* in pictures show low complexity regions

As discussed above and shown in Fig. 3, the novel mutation (p.N225M) is the underlying cause of a big frame shift and the loss of a large part of the deduced novel protein due to the early truncation which results in conformational instability. The modeling of the deduced novel mutation also reveals the structural importance of the cutting regions in dysfunctioning of the MMAA novel mutated protein. The MMAA protein contains the ArgK (PF03308) conserved domain which starts at p.101 and ends at p.384. Therefore a very large part of the protein lost (from p.225 to the end) contains the elements of this conserved domain site. The function of this domain is not completely known yet. However, it may have GTPase and ATPase activity and may also contain important binding sites of the MMAA protein due to its roles and conservation history. Moreover, the MMAA protein is a cobalamin cofactor which is considered to have a confident (vastly reported in the literature: data collected from www.string-db.org at String: 9606.ENSP00000281317) interaction with MUT protein. The *MUT* gene has a B12-binding site which can bind two different types of cobalamin cofactors, methylcobalamin and adenosylcobalamin, like the one described here [12–14]. It can thus be assumed that the loss of a large part of the MMAA protein (involving the conserved domain ArgK (PF03308) due to the novel mutation p.N225M) and the alteration of twelve amino acids (LYS p.226 to THR p.237) which happened due to the frame shift caused by c.674delA may drastically affect the binding activity of these two proteins. This effect can be considered as either removing the complete interaction sites or reducing the binding parts between them in such a way that the resulting proteins may fail to interact completely. Therefore in both situations the resulting interaction would not be completely functional.

## Discussion

In 1968, Rosenberg and Lindblad described infants with severe metabolic ketoacidosis and developmental delay with abnormally high levels of methylmalonate in their blood and urine. Treatment of these infants with AdoCbl reduced both the severity of their symptoms and methylmalonate excretion [15, 16]. Further studies indicated that these infants expressed normal methylmalonyl CoA mutase, but synthesis of AdoCbl was impaired [17, 18]. Kaye et al. (1974), reported two patients with MMA who expressed normal methylmalonyl CoA mutase, but were defective in AdoCbl synthesis and unresponsive to cyanocobalamin treatment. Biochemical and genetic analyses showed that cblB was also defective in these patients [19].

Since then, more than 45 different disease-causing mutations have been identified in MMAA, which include 30 missense or nonsense mutations, four splicing mutations, five small deletions, five small insertions, and one gross deletion. All the mutations are available on the Human Gene Mutation Database (HGMD) [20–24]. It is also very interesting to know that the MMAA protein has a low complexity region which starts at p.213 and ends at p.224, an amino acid before the reported novel mutation (p.N225M). Low complexity regions are prone to mutation as described previously [25] and in our case; the mutation occurred immediately after the end of this region. This low complexity region is demonstrated in both normal and novel mutated (p.N225M) MMAA protein in Fig. 3 above. Molecular analysis has not been performed on MMAA in the Iranian population. Early detection of MMA is critical; the girl in this family who was diagnosed at 2 months has survived, while the boy, who was diagnosed at 1.5 years, died soon after. Early diagnosis can prevent metabolic crises and mental retardation. Identification of mutations in MMA patients aids prenatal diagnoses and determination of the parents’ and relatives’ carrier status.

## Abbreviations

AdoCbl: Adenosylcobalamin
Cbl: Cobalamin
HGMD: Human Gene Mutation Database
MCM: Methylmalonyl-CoA mutase
MeCbl: Methylcobalamin
MMA: Methylmalonicacidemia
mRNA: Messenger RNA
MUMS: Mashhad University of Medical Sciences
PA: Propionic acidemia
SMTL ID: Swiss Model Template ID

## References

[1] Rosenblatt DS, Cooper BA (1987). Inherited disorders of vitamin B12 metabolism. Blood Rev.

[2] Rosenblatt DS, Fenton WA, Scriver CR, Beaudet AL, Valle D, Sly WS (2001). Inherited disorders of folate and cobalamin transport and metabolism. The Metabolic and Molecular Bases of Inherited Disease.

[3] Dobson CM, Wai T, Leclerc D, Wilson A, Wu X, Dore C (2002). Identification of the gene responsible for the cblA complementation group of vitamin B12-responsive methylmalonic acidemia based on analysis of prokaryotic gene arrangements. Proc Natl Acad Sci U S A.

[4] Korotkova N, Lidstrom ME (2004). MeaB is a component of the methylmalonyl-CoA mutase complex required for protection of the enzyme from inactivation. J Biol Chem.

[5] Miller SA, Dykes DD, Polesky HF (1988). A simple salting out procedure of extracting DNA from human nucleated cells. Nucl Acid Res.

[6] Arnold K, Bordoli L, Kopp J, Schwede T (2006). The SWISS-MODEL workspace: a web-based environment for protein structure homology modelling. Bioinform.

[7] Froese DS, Kochan G, Muniz JR, Wu X, Gileadi C, Ugochukwu E (2010). Structures of the human GTPase MMAA and vitamin B12-dependent methylmalonyl-CoA mutase and insight into their complex formation. J Biol Chem.

[8] Weiner S, Kollman P, Case D, Singh C, Ghio C, Alagona G (1984). A new force field for molecular mechanical simulation of nucleic acids and proteins. J Am Chem Soc.

[9] Li Z, Scheraga HA (1987). Monte Carlo-minimization approach to the multiple-minima problem in protein folding. Proc Natl Acad Sci U S A.

[10] Laskowski RA, MacArthur MW, Moss DS, Thornton JM (1993). PROCHECK: a program to check the stereochemical quality of protein structures. J App Cryst.

[11] Vriend G (1990). WHAT IF: a molecular modeling and drug design program. J Mol Graph.

[12] Krautler B (2005). Vitamin B12: chemistry and biochemistry. Biochem Soc Trans.

[13] Ludwig ML, Matthews RG (1997). Structure-based perspectives on B12-dependent enzymes. Annu Rev Biochem..

[14] Banerjee R, Ragsdale SW (2003). The many faces of vitamin B12: catalysis by cobalamin-dependent enzymes. Annu Rev Biochem..

[15] Rosenberg LE, Lilljeqvist A, Hsia YE (1968). Methylmalonic aciduria: metabolic block localization and vitamin B 12 dependency. Sci.

[16] Lindblad B, Lindblad BS, Olin P, Svanberg B, Zetterström R (1968). Methylmalonic acidemia. A disorder associated with acidosis, hyperglycinemia, and hyperlactatemia. Acta Paediatr.

[17] Mahoney MJ, Rosenberg LE, Harvey Mudd S, William UB (1971). Defective metabolism of vitamin B12 in fibroblasts from children with methylmalonicaciduria. Biochem Biophys Res Commun.

[18] Rosenberg LE, Lilljeqvist AC, Hsia YE, Rosenbloom FM (1969). Vitamin B12 dependent methylmalonic aciduria: defective B12 metabolism in cultured fibroblasts. Biochem Biophys Res Commun.

[19] Kaye CI, Morrow G, Nadler HL (1974). In vitro “responsive” methylmalonic acidemia: A new variant. J Pediatr.

[20] Yang X, Sakamoto O, Matsubara Y, Kure S, Suzuki Y, Aoki Y (2004). Mutation analysis of the MMAA and MMAB genes in Japanese patients with vitamin B(12)-responsive methylmalonic acidemia: identification of a prevalent MMAA mutation. Mol Genet Metab.

[21] Lerner-Ellis JP, Dobson CM, Wai T, Watkins D, Tirone JC, Leclerc D (2004). Mutations in the MMAA gene in patients with the cblA disorder of vitamin B12 metabolism. Hum Mutat.

[22] Martinez MA, Rincon A, Desviat LR, Merinero B, Ugarte M, Perez B (2005). Genetic analysis of three genes causing isolated methylmalonic acidemia: identification of 21 novel allelic variants. Mol Genet Metab.

[23] Merinero B, Perez B, Perez-Cerda C, Rincon A, Desviat LR, Martinez MA (2008). Methylmalonic acidaemia: examination of genotype and biochemical data in 32 patients belonging to mut, cblA or cblB complementation group. J Inherit Metab Dis.

[24] Guven A, Cebeci N, Dursun A, Aktekin E, Baumgartner M, Fowler B (2012). Methylmalonic acidemia mimicking diabetic ketoacidosis in an infant. Pediatr Diabetes.

[25] Lenz C, Haerty W, Golding GB (2014). Increased Substitution Rates Surrounding Low-Complexity Regions within Primate Proteins. Genome Biol Evol.

